# Retrospective study of 353 confirmed cases of urolithiasis in dogs and cats treated at veterinary clinics in the northern region of Pará, Brazil

**DOI:** 10.29374/2527-2179.bjvm005624

**Published:** 2025-03-13

**Authors:** Welligton Conceição da Silva, Bárbara Camila Ataíde Rocha, Simone Vieira Castro, Tatiane Silva Belo, Carlos Eduardo Lima Sousa, Fortunato Jerônimo Diniz Serruya, Raimundo Nonato Colares Camargo-Júnior

**Affiliations:** 1 Departamento de Medicina Veterinária, Centro Universitária da Amazônia (UNAMA), Santarém, PA, Brazil.; 2 Departamento de Medicina Veterinária, Instituto Federal de Educação, Ciência e Tecnologia do Pará, Santarém, PA, Brazil.; 3 Centro Universitário Católico do Tocantins, Palmas, TO, Brazil.

**Keywords:** urolithiasis, urolith, urinary vesicles, urolitíase, urólito, vesículas urinárias

## Abstract

Urolithiasis is the third most prevalent disease of the urinary tract of dogs and cats, and is characterized by presence of crystals and / or formation of stones. The present study objective was to report the confirmation rate of suspected cases of urolithiasis in dogs and cats treated at veterinary clinics in the city of Santarém, Pará. A retrospective cohort study was carried out from the database of medical records from six veterinary clinics from the years 2012 to 2023. Dogs and cats were evaluated for clinical suspicion, species, age, breed, feeding type, diagnosis, location of the urolith and type of urolith. As the data did not present a normal distribution, they were submitted to the Chi-square test and Fisher's exact test. The analyses were performed using Statistical Analysis Software (SAS), considering a 5% significance level. Of the 532 files analyzed of suspected cases, 353 were confirmed. The percentage of cases in cats (72.23%) was higher than in dogs (55.37%). The age range between 2 and 5 years was the period when it had more confirmed cases and most of the uroliths were found in the urinary vesicle, both in dogs and cats. It was concluded that there was a high index of confirmed cases of urolithiasis in dogs and cats, being superior in this last species. In addition, in both species are more affected animals between 2 and 5 years of age, being more common the location of uroliths in the bladder.

## Resumo

Urolitíase é a terceira doença mais prevalente do trato urinário de cães e gatos, e é caracterizada pela presença de cristais e/ou formação de cálculos. O objetivo do presente estudo foi relatar a taxa de confirmação de casos suspeitos de urolitíase em cães e gatos atendidos em clínicas veterinárias da cidade de Santarém, Pará. Foi realizado um estudo de coorte retrospectivo a partir do banco de dados de prontuários de seis clínicas veterinárias dos anos de 2012 a 2023. Cães e gatos foram avaliados quanto à suspeita clínica, espécie, idade, raça, tipo de alimentação, diagnóstico, localização e tipo do urólito. Como os dados não apresentaram distribuição normal, foram submetidos ao teste Qui-quadrado e teste exato de Fisher. As análises foram realizadas por meio do Statistical Analysis Software (SAS), considerando um nível de significância de 5%. Dos 532 prontuários analisados de casos suspeitos, 353 foram confirmados. O percentual de casos em gatos (72,23%) foi maior que em cães (55,37%). A faixa etária entre 2 e 5 anos foi o período em que houve mais casos confirmados e a maioria dos urólitos foi encontrada na vesícula urinária, tanto em cães quanto em gatos. Concluiu-se que houve alto índice de casos confirmados de urolitíase em cães e gatos, sendo maior nesta última espécie. Além disso, em ambas as espécies são mais acometidos animais entre 2 e 5 anos de idade, sendo mais comum a localização dos urólitos na bexiga.

## Introduction

Data coming from veterinary clinic centers on uroliths provide information on patient risk factors for uroliths, which are important for their clinical recognition and for understanding the pathophysiology (Hunprasit et al., 2019, Kopecny et al., 2021). Urolithiasis is a condition resulting from metabolic alterations that occur commonly in the medical routine of small animals, affecting 15-23% in felines (Jepson, 2023; Putchakayala & Haritha, 2024; Ximenes et al., 2023) and about 33% in canines (Naeverdal et al., 2023; Mulyani et al., 2024; Yaygıngül, 2024). The clinical prevalence of urolithiasis in dogs ranges from 0.5% to 1% (Elkewahy et al., 2023). It is a condition that has high rates of recurrence (To et al., 2024).

It is considered the second most common cause of feline lower urinary tract disease (FLUTD) (with 15 to 20%), with the first being idiopathic cystitis (with 55 to 65%), followed by anatomical abnormalities and behavioral problems (with about 10%) of the reports (Gülersoy et al., 2023). It can be said that approximately 13% of the causes of urinary tract disorders in cats and 18% in dogs are represented by urolithiasis. (Mustafa et al., 2023).

This disease is defined as the formation of stones or uroliths and can be present in the kidneys, ureter, urinary vesicle, or urethra (Kim & Oh, 2023). Uroliths are polycrystalline concretions composed primarily of organic and inorganic crystalloids and smaller amounts of organic matrix, formed by aggregates of urinary solutes, precipitated and organized in a central nucleus (nest or crystal nucleus) which, in turn, is surrounded by concentric laminae and surface crystals (Monnet, 2023; Santos et al., 2023).

Uroliths are classified according to their mineral composition, location, and shape, with struvite, calcium oxalate, urate, mixed, silicate, and cystine being the main ones found in dogs and cats (Stavroulaki et al., 2024). Regarding its incidence, dogs are more predisposed to develop calcium oxalate stones, and cats to struvite stones (Bartges & Corbee, 2023).

Several factors can lead to the formation of uroliths, such as changes in urinary pH, infections, high concentration of crystalloids in urine, reduced water consumption, as well as a decrease in urinary frequency associated with supersaturation of urine with salts and combined with a high supply of minerals and proteins in the diet (Cook, 2023; Eicher et al., 2023; Geddes et al., 2023; Gomes et al., 2018). Acquired lesions, inherited anatomical alterations, such as bladder diverticulum, and genetic factors that predispose the animal to urinary infections that can lead to the formation of struvite uroliths, locality, and time can also influence the formation of uroliths (Kant et al., 2023; Xu et al., 2024).

Clinical features depend on the number, type, and location of uroliths in the urinary tract. The most observed clinical signs are frequency, dysuria, stranguria, hematuria, urinary incontinence and uremia, ischuria and in cases of complications, they may present progression to urethral and/or bladder obstruction, hydroureter and hydronephrosis, bladder or urethral rupture, pyelonephritis, urethritis and bladder dilation. Systemic signs such as diarrhea, anorexia, vomiting, and lethargy may occur, in addition to the presence of palpable urinary gallstones (Ardebili & Razavi, 2023; Gloor et al., 2024; Ozcan et al., 2023).

The diagnosis of urolithiasis involves the patient's history, physical examination, laboratory findings, and imaging tests such as ultrasound and radiography (Ji et al., 2023; Malhi et al., 2023; Montatore et al., 2023). In view of the high incidence of cases of urolithiasis among urinary tract disorders in the clinical medical routine of small animals, the aim of this study was to report the confirmation rate of suspected cases of urolithiasis in dogs and cats treated at private veterinary clinics in the municipality of Santarém, Pará.

## Material and methods

### Study population

A retrospective cohort study was carried out based on the analysis of the database of six randomly chosen veterinary clinics, located in the municipality of Santarém (02º 26' 35" S and 54º 42' 30" W), mesoregion of Baixo Amazonas, northern region of Pará. Medical records were evaluated between January 1, 2012 and December 31, 2023, separating patients specifically with urinary tract disorders (lower and upper), with suspected urolithiasis.

### Medical record data extracted

A total of six veterinary clinics (A-E) were consulted, the clinics were in different neighborhoods, with a total of 532 suspected cases from all the clinics, broken down by establishment (clinics A - 102 cases, B - 112 cases, C - 113 cases, D - 108 cases, E - 97 cases). Information was collected such as: year of presentation of the urolith, species (canine or feline), age (between 6 months and 1 year (puppies), 1 to 2, 2 (youngsters) to 3, 3 to 5 and over 5 years (adults)), breed (all breeds recorded in the medical records), type of food provided (kibble and kibble with homemade food), as well as clinical suspicion, diagnostic confirmation by radiography or ultrasound (total number of suspects) and location of the urolith. The type of urolith that affected both dogs and cats was also observed. The medical records showed no comorbidities in the animals studied.

During the anamnesis, most of the medical records described that the main complaints of the owners of canines and felines with suspected urolithiasis were difficulty in urinating (dysuria), increased urination but in small quantities (polyuria), the presence of blood (hematuria), excessive licking of the genital area, apathy, inappetence, straining to urinate and abdominal pain.

### Statistical analysis

For the statistical analysis, the data were submitted to the Shapiro-Wilk test to verify the normality and homoscedasticity of the data. As the data did not present a normal distribution, they were submitted to the Chi-square test and Fisher's exact test. The analyses were performed using Statistical Analysis Software (SAS), considering a 5% significance level.

## Results

**Figure 1:**
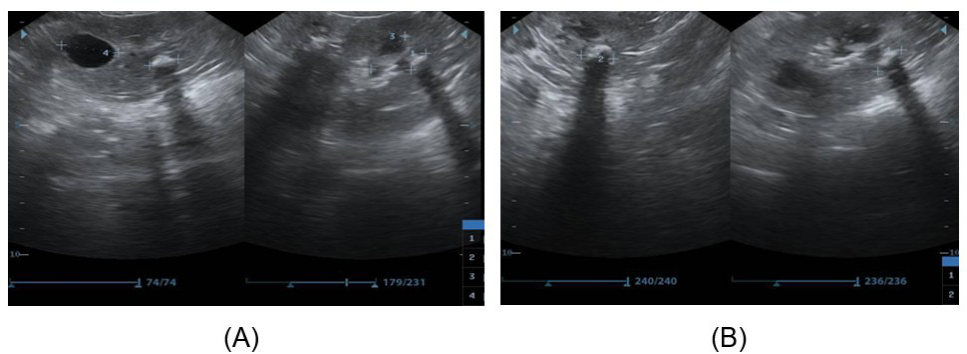
Ultrasound image of a kidney containing uroliths diagnosed in dogs and cats in veterinary clinics in the northern region of Pará. In (a) left kidney. In (b) right kidney.

Of the 532 suspected cases analyzed, 353 were confirmed, making a confirmation rate of 66.35%. Analyzing the clinical suspicions, it was identified that 351 (65.97%) presented bleeding through the urethral canal as one of the most common clinical signs of urinary tract disorders, and the remaining 181 (34.03%) suspected cases already had a history of urinary tract infection. Of the confirmed cases, the presence of stones was detected by ultrasonography in 351 animals (Figure 1) and only two with radiography.

It was observed that the total number of confirmed cases among the species, most were cats with 255 (71.83%) (p<0.05), however, it should be considered that the number of cats with suspected 355 cases is higher than that of suspected cases in dogs (177). In dogs, the percentage of confirmed cases in each year did not differ (p<0.05), with the exception of 2016 and 2017, where a low confirmation rate was observed. In cats, the percentage of occurrence was lower in the years 2014 and 2018, however, there was no statistical difference from the years 2012, 2013, 2015, 2016 and 2017 (p>0.05). However, of the confirmed cases among the different species in the same year, a higher percentage of confirmation was observed in cats from 2016 to 2023 when compared to dogs (p<0.05).

**Figure 2:**
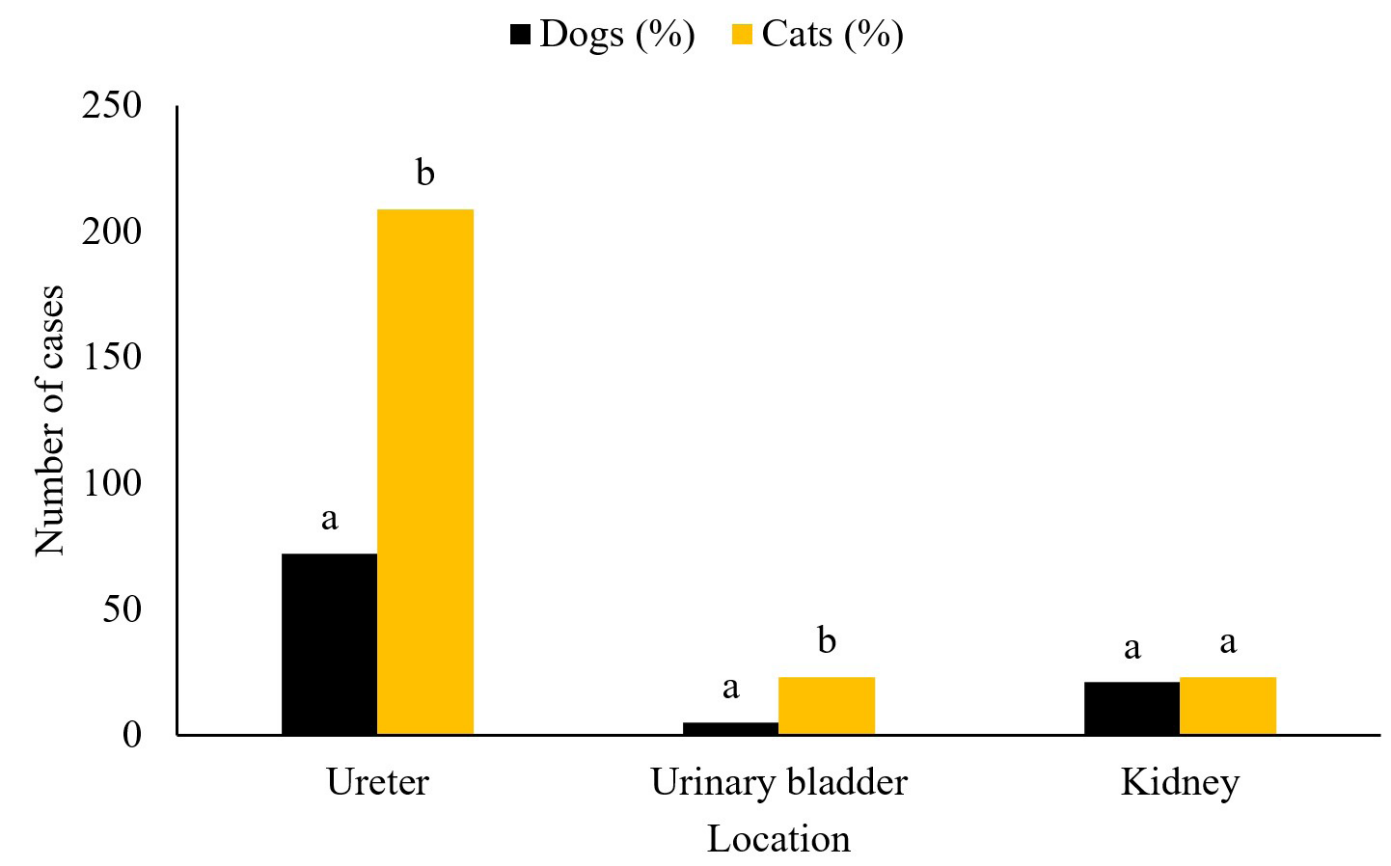
Location of uroliths diagnosed in dogs and cats in veterinary clinics in the northern region of Pará. Note: a, b: indicates statistical difference between lines; *differs statistically between species of the same age.

Regarding the location of the urolith in the urinary tract, it was found in dogs that most of them were in the bladder, followed by the kidneys with 22% and in only 5% of the cases in the urethra (Figure 2). In cats, a higher number of uroliths was observed in the urinary vesicle and equal percentages in the kidneys and urethra (Figure 2).

**Figure 3:**
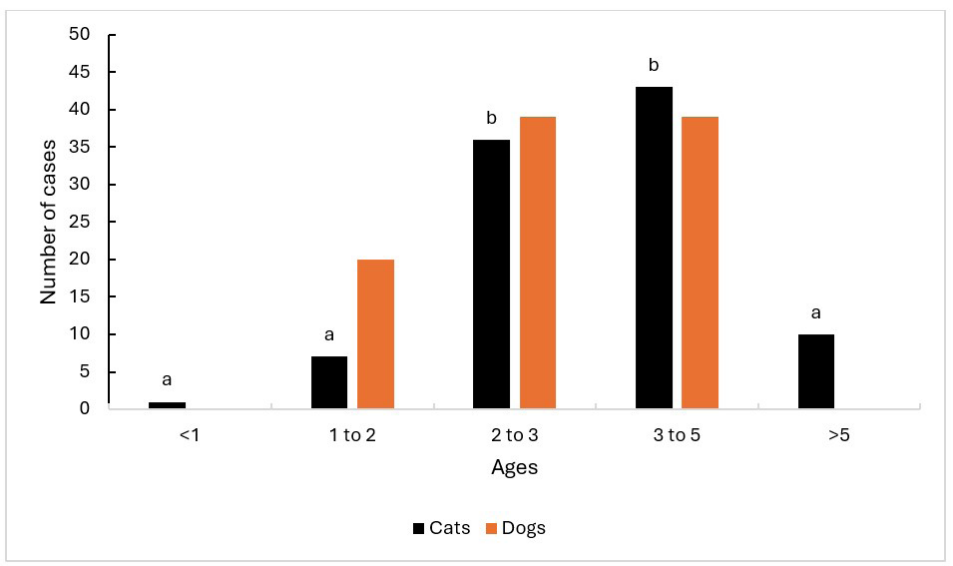
Distribution of confirmed cases of uroliths in dogs and cats according to age group. Note: a, b or ab: indicates statistical difference between lines; *differs statistically between species of the same age.

It was found that most of the animals had urolithiasis in adulthood, corresponding to between 3 and 5 years (p<0.05), with 43.05% of the cases. Regarding the age of the affected animals, a high rate of confirmed cases was found in both dogs and cats in the 2 to 3 years and 3 to 5 year groups (p<0.05), being significantly higher than in the other age groups (Figure 3).

**Figure 4:**
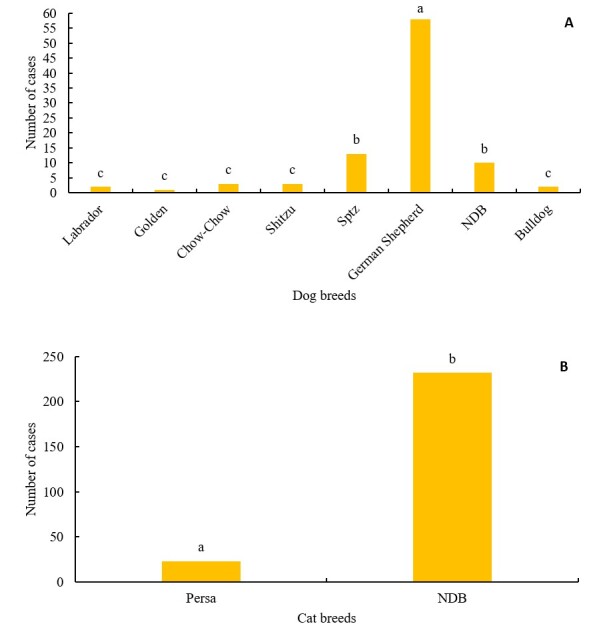
Number of cases of dogs (A) and cats (B) with confirmed cases of urolithiasis in veterinary clinics in the northern region of Pará. Note: NDB = no defined breed. Note: a, b indicates statistical difference between lines.

Of the total number of animals that presented urolithiasis in this study, the mixed breed animals were the ones that stood out the most, however, it should be noted that the number of mixed breed animals was much higher than those with defined breeds, such as Labradors, Golden, Chow-chows, Shitzu, German Spitz (SPTZ), German Shepherds and Bulldogs (Figure 4a, b). There was no difference between dogs and cats fed only with kibble or kibble plus homemade food (p>0.05).

**Figure 5:**
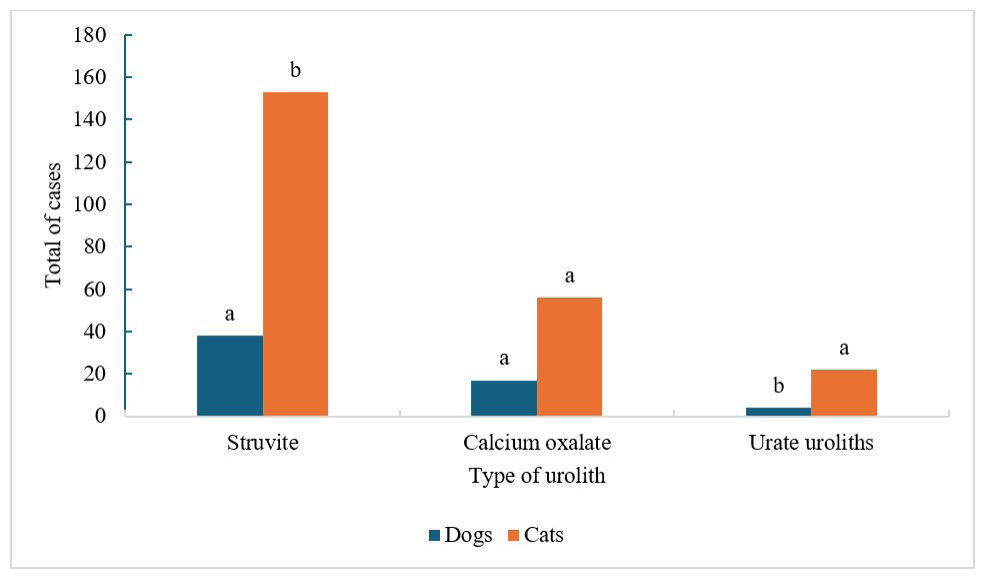
Types of uroliths diagnosed in dogs and cats. Note: a, b indicates statistical difference between lines.

Of the total of 353 confirmed cases, urolith types were diagnosed in 60.20% of cases in dogs and 90.58% in cats. Of these, the highest numbers of struvite-type calculi were observed, followed by calcium oxalate and urate (p<0.05) (Figure 5).

## Discussion

The number of suspected cases of urolithiasis in cats increased over the years 2012 to 2023, this can be attributed to the possible increase in the demand for veterinary medical service by owners (Joubran et al., 2024). In addition, a higher number of confirmed cases was found in cats than in dogs (p<0.05). This result may be related to the strong concentration of cat urine, which, because they originate from desert regions, have adapted to consume little liquid, and consequently produce little urine volume, making them more susceptible to the development of urolithiasis (Defarges et al., 2020; Hall et al., 2021; Queau et al., 2020).

It should be noted that stones can form in any region of the urinary tract of animals (Hoelmer et al., 2022), and the dog has a higher incidence in the bladder (Kopecny et al., 2021). Grauer (2010) and Inkelmann et al. (2012) agree that about 95% of uroliths in dogs are found in the urinary gallbladder.

Neta and Munhoz (2008) mention that only 5% to 10% of uroliths are located in the kidneys or ureters, and even though the kidney is one of the least common sites, it showed relevance in terms of percentage (22%), and even compared to the percentage in cats (9%). Kaufmann et al. (2011) mentions that in felines, uroliths are found more in the urinary vesicle or urethra, with the ureters and kidneys being the least common sites, corroborating the results obtained in this study.

Uroliths in the upper urinary tract are not commonly reported in dogs and cats. This is because there is a hypothesis that there may be a correlation between the positioning of the kidney and urinary vesicle in relation to gravity in quadrupeds and biped. In quadrupedal animals, the horizontal positioning of the kidneys and bladder are aligned parallel to the ground, causing gravity to influence their anatomy and physiology, allowing urine to flow through the ureters, reducing the occurrence of urinary stasis and the formation of stones in the upper urinary tract. On the other hand, in bipedal animals that remain upright, gravity has an impact on the accumulation of sediment and the formation of stones in the kidneys and ureters, since they are located above the bladder. This anatomical and consequently physiological difference explains the lower incidence of uroliths in the upper urinary tract in quadrupeds when compared to bipeds (Kovaříková et al., 2021).

In view of the above, according to Neta and Munhoz (2008), Breshears and Confer (2017) and Silva et al. (2022), uroliths that do not obstruct can persist for years without causing lesions or being clinically noticed. Kaufmann et al. (2011) cite that in the canine species, small uroliths can remain asymptomatically in the urinary tract for months to years.

In a study in the United Kingdom, cases were observed for the average ages of 7.0 years in dogs and 7.4 years in cats (Stavroulaki et al., 2024), however, in a study in Mexico City, it was shown that the age of the animals ranged from 4 months to 14 years, with an average of 5 years (Mendoza-López et al., 2019). Inkelmann et al. (2012) at the Department of Pathology of the Federal University of Santa Maria (LPV-UFSM), performed an analysis among 76 dogs with urolithiasis, and found that most were adults, with an average age of 5 years, corroborating the results of this study that demonstrated that age (between 3 and 5 years) is the period in which there were more cases of urolithiasis.

According to a study carried out by Oyafuso et al. (2010) by quantitative evaluation of the composition of 156 dog uroliths at the Veterinary Hospital of the Faculty of Veterinary Medicine and Animal Science of USP - São Paulo, mixed breeds were more affected, corroborating the results of this work, however, in a study carried out with 76 dogs by Inkelmann et al. (2012) at the Department of Pathology of the Federal University of Santa Maria (LPV-UFSM), Most of the dogs with defined breed (56.6%) had urolithiasis.

The total number of Shitzu dogs that had urolithiasis, which according to Stavroulaki et al. (2024) represents one of the main breeds affected by the presence of uroliths, as well as the Miniature Schnauzer, Lhasa apso, Yorkshire terrier, Bichon frisé and Poodle. Regarding the cats, although of reduced number, all of the Persian breed had the suspicion of urolithiasis confirmed. This breed of cat, according to Neves et al. (2011) is one of the most predisposed to this condition, as well as the Russian Blue and Himalayan breed.

It should be considered that diet may not influence the formation of uroliths, as the development of urolithiasis is related to dietary and non-dietary factors (Kachkoul et al., 2023), as well as factors linked to obesity, castration status, sex, age, and race (Burggraaf et al., 2021).

The data shows that the majority of confirmed cases of urolithiasis in dogs and cats are more often struvite-type stones, which are frequently associated with urinary infections, with diet being the main factor due to the change in pH making it more alkaline (Parmar et al., 2021; Yaygıngül, 2024). In cats, urinary infections occur less frequently, but a diet rich in magnesium and reduced water intake contribute to stone formation (Gomes et al., 2018). The prevalence of this type of stone suggests that the main cause is related to dietary factors and bacterial infections of the urinary tract, thus favoring the diagnosis, management and prevention of urolithiasis, especially in canines, which are often more affected.

In addition, calcium oxalate stones are the second most frequently observed type, reflecting the need to establish a balance between the metabolism of minerals such as calcium and oxalate levels, influenced by the concentration present in diets and also by genetic conditions (Bartges, 2016; Grauer, 2015). Furthermore, urate stones were also one of the most common occurrences during the study, especially in canines of predisposed breeds such as Dalmatians or hepatopathic animals. There is therefore a need to develop more species-specific preventive and therapeutic approaches, taking into account various factors such as diet, metabolism, genetics and infection.

## Conclusion

There was a high rate of confirmed cases of urolithiasis in both dogs and cats, being higher in the latter species. Both species are most affected in animals between 2 and 5 years of age, and the location of uroliths in the urinary gallbladder is more common. In addition, the results obtained in this study help veterinary professionals on the epidemiology of urolithiasis in dogs and cats.
